# Chloroplast Genome of the Folk Medicine and Vegetable Plant *Talinum paniculatum* (Jacq.) Gaertn.: Gene Organization, Comparative and Phylogenetic Analysis

**DOI:** 10.3390/molecules23040857

**Published:** 2018-04-09

**Authors:** Xia Liu, Yuan Li, Hongyuan Yang, Boyang Zhou

**Affiliations:** State Key Laboratory of Food Nutrition and Safety, Key Laboratory of Food Nutrition and Safety, Ministry of Education of China, College of Food Engineering and Biotechnology, Tianjin University of Science & Technology, No. 29, 13th Street, TEDA, Tianjin 300457, China; 13820282933@163.com (Y.L.); yanghongyuan1218@sina.com (H.Y.); zhouboyang456@163.com (B.Z.)

**Keywords:** *Talinum paniculatum*, chloroplast genome, medicinal plant, phylogeny

## Abstract

The complete chloroplast (cp) genome of *Talinum paniculatum* (Caryophyllale), a source of pharmaceutical efficacy similar to ginseng, and a widely distributed and planted edible vegetable, were sequenced and analyzed. The cp genome size of *T. paniculatum* is 156,929 bp, with a pair of inverted repeats (IRs) of 25,751 bp separated by a large single copy (LSC) region of 86,898 bp and a small single copy (SSC) region of 18,529 bp. The genome contains 83 protein-coding genes, 37 transfer RNA (tRNA) genes, eight ribosomal RNA (rRNA) genes and four pseudogenes. Fifty one (51) repeat units and ninety two (92) simple sequence repeats (SSRs) were found in the genome. The pseudogene *rpl23* (Ribosomal protein L23) was insert AATT than other Caryophyllale species by sequence alignment, which located in IRs region. The gene of *trnK-UUU* (tRNA-Lys) and *rpl16* (Ribosomal protein L16) have larger introns in *T. paniculatum*, and the existence of *matK* (maturase K) genes, which usually located in the introns of *trnK-UUU*, rich sequence divergence in Caryophyllale. Complete cp genome comparison with other eight Caryophyllales species indicated that the differences between *T. paniculatum* and *P. oleracea* were very slight, and the most highly divergent regions occurred in intergenic spacers. Comparisons of IR boundaries among nine Caryophyllales species showed that *T. paniculatum* have larger IRs region and the contraction is relatively slight. The phylogenetic analysis among 35 Caryophyllales species and two outgroup species revealed that *T. paniculatum* and *P. oleracea* do not belong to the same family. All these results give good opportunities for future identification, barcoding of Talinum species, understanding the evolutionary mode of Caryophyllale cp genome and molecular breeding of *T. paniculatum* with high pharmaceutical efficacy.

## 1. Introduction

Chloroplasts are the metabolic centers of our world due to the crucial role of converting sunlight into energy and releasing oxygen. Chloroplast genome encodes many key proteins that are involved in photosynthesis and other important metabolic processes for plant interactions with their environment, such as drought, salt and light [1,2,3,4]. The availability of over 2400 sequenced chloroplast genomes (http://www.ncbi.nlm.nih.gov/genomes/) gave us insights to understand the plant biology diversity, evolution, climatic adaptation, DNA barcoding and expression high-value agricultural or biomedical products by genetic engineering [1,2,5,6,7,8,9].

Java ginseng (*Talinum paniculatum* (Jacq.) Gaertn.), one of the most popular medicinal plants, belongs to the genus *Talinum* Adans. in the Portulacaceae family of the Caryophyllales. It is widely used for many ailments, including cancer, diabetes, hepatic disorders, leishmaniasis and reproductive disorders due to its high concentrations of alkaloids, flavonoids, tannins, steroids, and triterpenes [10,11,12,13,14]. The root of *T. paniculatum* has lots of valuable components, which are similar to those in *Panax* [15]. Meanwhile, the leaves of *T. paniculatum* are widely used as a green leafy vegetable for human consumption in many parts of South America, Africa and Asia. 

Like *Portulaca oleracea*, *T. paniculatum* has high adaptability to drought, salinity, and low nutrient conditions [16]. Few reports have been published on the genetic diversity of chloroplast DNA from the Portulacaceae. The relationship of *Portulaca* to Anacampserotaceae, Cactaceae Juss, and Talinaceae is still uncertain [17,18,19,20]. The Portulacaeae are composed only a single genus of *Portulace* [21]. Molecular barcodes based on the chloroplast genome have shown great potential for species discrimination, especially between closely related taxa, but to date, chloroplast genome sequences of only two genus of the Portulacaceae have been analyzed, which are *Carnegiea gigantean* and *Portulaca oleracea* [22,23,24]. At both the species and population levels, the complete chloroplast genome sequence might enhance our ability to explore reliable barcoding for accurate plant identification [6,7,25].

Here, we report for the first time the complete cp genome sequence of *T. paniculatum*. Meanwhile, the gene structure and organization of the cp genome were analyzed. Besides, the cp genome sequences was compared with other genus of Caryophyllales. We also present the results of phylogenetic analyses of DNA sequences for 48 protein-coding genes from *T. paniculatum*, *35* Caryophyllales cp genomes and two outgroups. 

## 2. Results

### 2.1. Features of the T. paniculatum cp Genome

**Figure 1:**
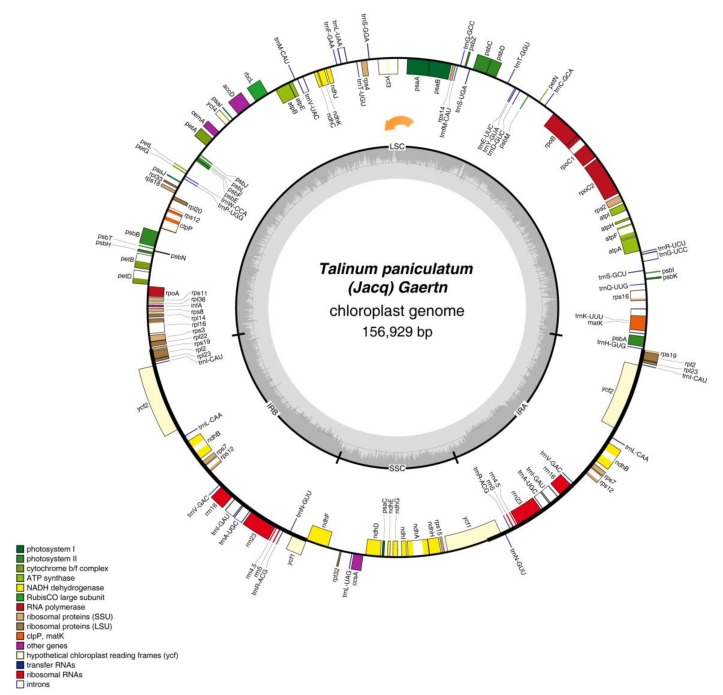
The complete chloroplast genome map of *Talinum paniculatum* (Jacp) Gaertn. Genes are color-coded based on functional group. Genes drawn inside the circle are transcribed clockwise, and those outside are transcribed counterclockwise. The genome orientation are the orange arrow.

The chloroplast genome of *T. paniculatum*, a typical quadripartite structure, is 156,929 bp in size, including a LSC region of 86,898 bp ranging from *trnH-GUG* to *rps19* (Ribosomal protein S19), a SSC region of 18,529 bp from *ndhF* (NAD(P)H dehydrogenase) to *ycf1* (hypothetical protein 1 gene), a pair of IR regions of 25,751 bp from *rps19* to pseudogene *ycf1* and ranging from *ycf1* to *rps19*, respectively (Figure 1 and Table 1). The GC content of cp genome in the cp total, LSC (large single copy), SSC (small single copy), IRA (inverted repeat A region) and IRB (inverted repeat B region) is 36.79%, 34.6%, 30.5%, 42.7% and 42.7%, respectively (Table 1), which is similar to the cp genome of other Caryophyllales [23,24] and the higher GC content in IR regions. A higher AT representation at the third codon position and second codon position was significant, which is one of the common characteristics of the chloroplast genome [26,27,28,29]. The overall cp genome of *T. paniculatum* is made up of 49.98% protein-coding regions, 5.76% rRNAs, 1.78% tRNAs and 50.02% non-coding regions.

In total, there are 113 unique functional genes and four pseudogenes in the chloroplast gene of *T. paniculatum* (Table 1 and Table 2). Among 113 functional genes, 79 protein-coding genes, 30 distinct tRNAs and four distinct rRNAs were contained (Table 2). Remarkably, because all rRNA, seven tRNA, four protein-coding genes and one pseudogene are replicated in the IR regions, the total genes in Figure 1 and Table 2 are 132. Fifty six protein-coding and 22 tRNA genes are located in LSC region, but only 12 protein-coding genes and one tRNA are located in the SSC region. Meanwhile, all eight rRNA are sited in IR regions, which has been found to be common in most cp genomes [23,30]. The *ycf1* gene is located at the IR and SSC boundary region, the *rps19* gene was located at the IR and LSC boundary region. Pseudogenes of *rpl23*, *rps19*, *ycf1* are all located in IR regions.

Among the 113 unique genes in *T. paniculatum* cp genome, 17 genes contain introns, including six tRNA genes and 11 protein-coding genes. Most of them only contain one intron, while like to other angiosperms, *ycf3*, *clpP* and *rps12* have two introns (Table 3). Twelve genes with introns are located in the LSC, four genes with introns are located in IR regions, and only one gene with intron is located in the SSC region. Interestingly, the 3′ exon of *rps12* is duplicated in IRs, but its 5′ exon shared by the 3′ exon was located in the LSC region. Consistent with many research results, the *matK* gene was inserted into the intron of *trnK-UUU*, so the intron of *trnK-UUU* became so maximal [23,30]. Comparing these 17 introns with *P. oleracea*, most of them is shorter in *T. paniculatum*, whereas the introns of *petB*, *petD* are larger, and *rpoC1* intron is the same size [23].

### 2.2. Codon Usage of the T. paniculatum cp Genome

As shown in Table 4, total 26,146 codons involved in the protein-coding in *T. paniculatum*. Among 26,146 codons, the amino acids used most frequently were leucine and isoleucine, which encode in 2780 (10.63%) and 2258 (8.63%) codons, respectively. While only 303 (1.50%) codons encode cysteine as the least frequent universal amino acid (Table 4).

The relative synonymous codon usage (RSCU) has been divided into four models, including lack of bias (RSCU < 1.0), low bias (1.0 < RSCU< 1.2), moderately biased (1.2 < RSCU< 1.3) and highly biased (RSCU > 1.3) [30,31]. As shown in Table 4, there are 32 lack of bias codons with values < 1.0, except tryptophan and methionine, four low bias codons, four moderately biased codons and 21 highly biased codons, respectively. The TAA stop codon was found to be preferred. Meanwhile, due to 29 preferred synonymous codons (RSCU > 1.0) end with A or T, the third position of CDS contains 70.5% A or T bases, a significantly higher percentage than in the 2nd position (62.1%) or the 1st position (54.4%) (Table 1 and Table 4). The results showed that the bias was significant in usage of synonymous codons except tryptophan and methionine in T. paniculatum as well as A/T ending rich in cp genome. These results revealed that the RSCU and codon usage exsited biased codon usage, which was consistent with previous reports in cp genomes of higher plants [32,33].

### 2.3. Repeats Structure and SSR in T. paniculatum cp Genome

**Figure 2:**
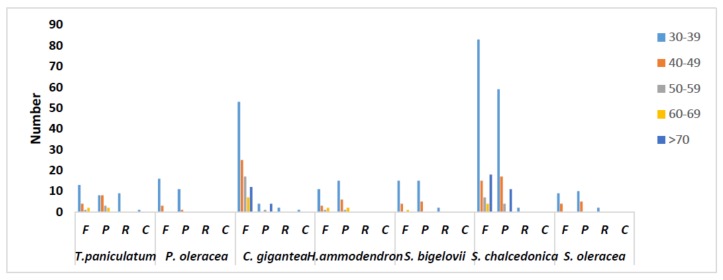
Repeat sequences in seven chloroplast genomes of Caryophyllales. REPuter was used to identify repeat sequences with length ≥30 bp and sequence identify ≥90% in the chloroplast genomes. F, P, R and C indicate the repeat types forward, palindrome, reverse and complement, respectively.

Analysis of the repeat structure analysis using REPuter detected a total of 51 repeat sequences, including 20 forward repeats, 21 palindromic repeats, one complement repeats and nine reverse repeats in the cp genome of *T. paniculatum* (Table 5). The repeats range from 30 to 61 bp in length and are mostly located in the intergenic spacer (IGS) and intron sequences. Twenty five repeats are located in exons of *matK*, *trnS-GGA*, *trnG-UCC*, *psaB*, *psaA* and *ycf2* genes, respectively. A comparative analysis revealed that 30–39 bp were the most repetitive in cp genome of seven species in Caryophyllales (Figure 2). Complement repeats were rare, as only *T. paniculatum* and *C. gigantea* contain them [24]. The reverse repeats of *T. paniculatum* are the most in the seven cp genomes.

**Figure 3:**
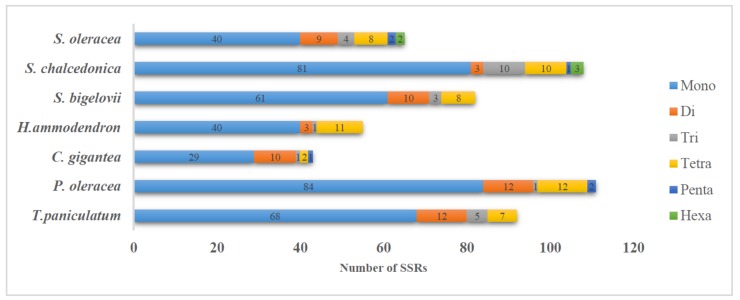
Distribution of SSRs present in seven chloroplast genomes of Caryophyllales.

There are 92 simple sequence repeats (SSRs) in the *T. paniculatum* chloroplast genome, the majority of which were mononuclear repeats (68). Twelve dinucleotide repeats, five trinucleotide repeats and seven pentanucleotide repeats were also found in *T. paniculatum* cp genome (Table 6, Figure 3). Whereas, only seven SSRs located in the CDSs (Table 7), including four mononucleotide, one dinucleotide and two pentanucleotide SSRs. The results indicate that all of the SSRs in CDs are located in the LSC region and most of them are AT repeats, which is consistent with the view that SSRs in cp genomes contribute to AT richness [32]. 

### 2.4. matK and rpl23 Diversity Analysis

**Figure 4:**
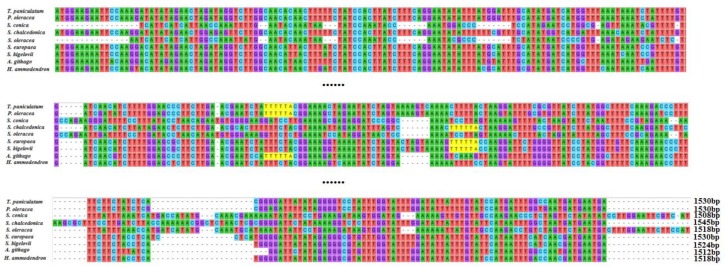
Sequence alignment of *matK* among nine species cp genome in Caryophyllales. As the *matK* gene are too long, only the sequences with greater variation were shown here.

The chloroplast genes *matK* and *rpl23* of *T. paniculatum* were compared with those of nine other Caryophyllaceae species. The *matK* sequence is often used in DNA barcoding, phylogenetic and evolutionary studies [34]. The sequences of *matK* genes of chloroplast DNA in *T. paniculatum*, *P. oleracea*, *S. conica*, *S. chalcedonica*, *S. europaea*, *S. bigelovii*, *A. githago*, *H. ammodendro and S. oleracea* show significant diversity by alignment analysis (Figure 4), while most of the *matk* (*T. paniculatum*, *P. oleracea*, *S. conica*, *S. chalcedonica*, *S. europaea*, *S. bigelovii*, *A. githaggo*, *H. ammodendro and S. oleracea*) were inserted into the introns of *trnK-UUU* [35]. Oddly, the *matK* gene is a pseudogene in *S. conica*, which has the shortest length [36]. The multiple alignment result revealed that the *matK* gene of *T. paniculatum* was relatively similar to that of *P. oleracea*, except for an AATT insert and eight single nucleotide polymorphisms (SNPs) (Figure 4). The *matK* genes from *S. chalcedonica* and *A. githago* displayed relatively high sequence similarity, due to the fact they both belong to the Sileneae in the Caryophyllaceae family [37]. The *matK* in *S. bigelovii* and *S. europaea* had high sequence similarity too, because they belong to the same family of Chenopodiaceae (Figure 4). These results revealed that the closer the relationship between species, the higher the sequence similarity of *matK*. Hence, consistent with many research results, the *matK* gene was one of the best choices for DNA barcoding, phylogenetic and evolutionary analysis [38,39,40].

**Figure 5:**
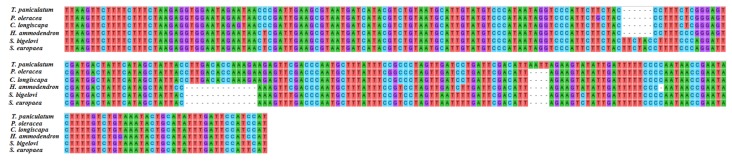
The gene of *rpl23* sequence alignment among six Caryophyllales species.

Ribosomal protein L23 (*rpl23*) is a protein component of the 60S large ribosomal subunit and is also a negative regulator of cellular apoptosis in animals [41]. The alignment of *rpl23* was carried out in *T. paniculatum*, *P. oleracea*, *C. longiscapa*, *S. conica*, *H. ammodendron*, *S. bigelovii*, *S. europaea* (Figure 5). The results revealed that *rpl23* gene of *T. paniculatum* was a pseudogene as in *H. ammodendron*, *S. bigelovii* and *S. europaea*. The *rpl23* gene lacked the CTTGACACCAAAGA sequence in *H. ammodendron*, *S. bigelovii* and *S. europaea*. However, the *rpl23* of *T. paniculatum* had AATT inserted. Interestingly, the *rpl23* was not presented in *A. githago*, *S. chalcedonica* and *A. hypochondriacus* [37]. The *rpl23* were normal genec in *P. oleracea* and *C. longiscapa*, which had some SNPs between them [42]. 

### 2.5. Comparative Analysis of the Chloroplast Genomes of the Caryophyllales

**Figure 6:**
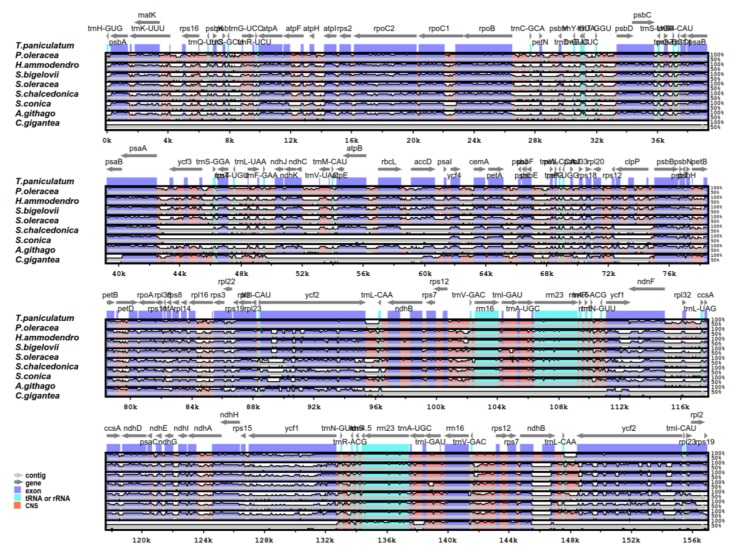
Complete chloroplast genome sequence comparison of eight species using mVISTA, with *T. paniculatum* as a reference. The horizontal axis corresponds to the coordinates within the chloroplast genome. The vertical scale represents the identity percentage. The grey lines and the arrows show the genes with their orientation and position. CNS: conserved noncoding sequences.

The genome sequence of *T. paniculatum* was compared with eight species of Caryophyllales using the mVISTA software (Figure 6). *T. paniculatum* had the biggest cp genome with the biggest IR region (25,751 bp), while *C. gigantea* had the smallest cp genome with one IR region loss [24]. The length differences among them were mainly caused by deletions in the non-coding regions. Meanwhile, the results showed that the non-coding region variation was significantly higher than that of the coding regions, and the IR region was more conserved than the LSC and SSC regions [43,44]. The *matK*, *accD*, *ndhF*, *infA*, *trnS-GGA*, *trnT-UGU*, *trnL-UAA*, *trnG-UCC*, *ycf2*, *ycf1*, *rpl23*, *ccsA*, *ndhG* and *rps19* genes were the most divergent coding regions. The introns of *ycf3*, *ndhA*, *rpl16*, *clpP*, *petB*, *trnK-UUU*, *rpoC*, *pet D* were relatively highly divergent, too.

### 2.6. IR Expansion and Contraction

**Figure 7:**
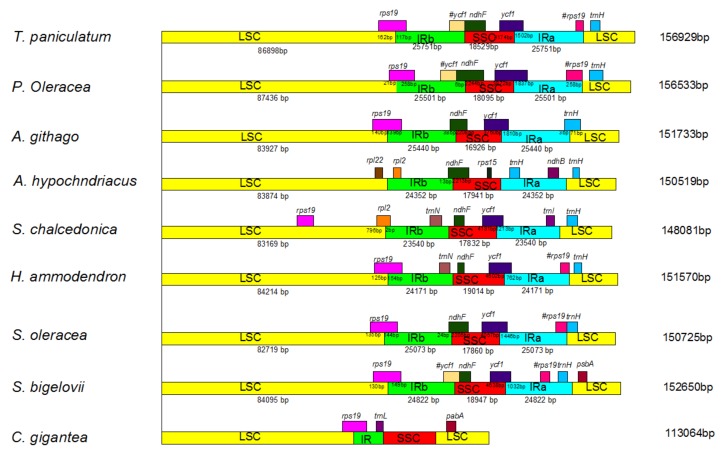
Comparison of the borders of the LSC, SSC and IR regions in nine Caryophyllales species. #indicates that the gene is a pseudogene.

IR contraction and expansion of *T. paniculatum* was analyzed by comparing the LSC/IRb/SSC/IRa boundary regions with seven families in the Caryophyllales (Figure 7). Among them, the chloroplast genome of *T. paniculatum* was the longest. The *ndhF* gene of *T. paniculatum* did not cross the IRb region and the SSC region, compared to *P. oleracea*, *A. githago*, *A. hypochndriacus* and *S. oleracea*, however it was the largest IR region among them (Figure 7). The *rps19* gene in the *T. paniculatum* cp genome was shifted by 117 bp from LSC to IRb at the LSC/IRb border, which was the smallest shift length compared to *P. oleracea*, *A. githago*, *H. ammodendron*, *S. oleracea and S. bigelovii* (Figure 7). Differently from other LSC/IRb border, the *rps19* gene of *S. chalcedonica* was entirely located in the LSC region. Unlike most LSC/IRa borders, the *trnH* gene of *S. bigelovii* was located in the LSC region, and the *trnH* of *T. paniculatum* was located in the LSC region as most of them. Oddly, a significant bigger IR size but the smallest *rps19* and *ycf1* length in the IR region were found in *T. paniculatum*. This phenomenon may be for two reasons: on one hand, pseudogenes exist, and on the other hand, there is a low occurrence of contraction by the fragment deletions in the intergenic regions in *T. paniculatum* [32].

### 2.7. Phylogenetic Analysis

**Figure 8:**
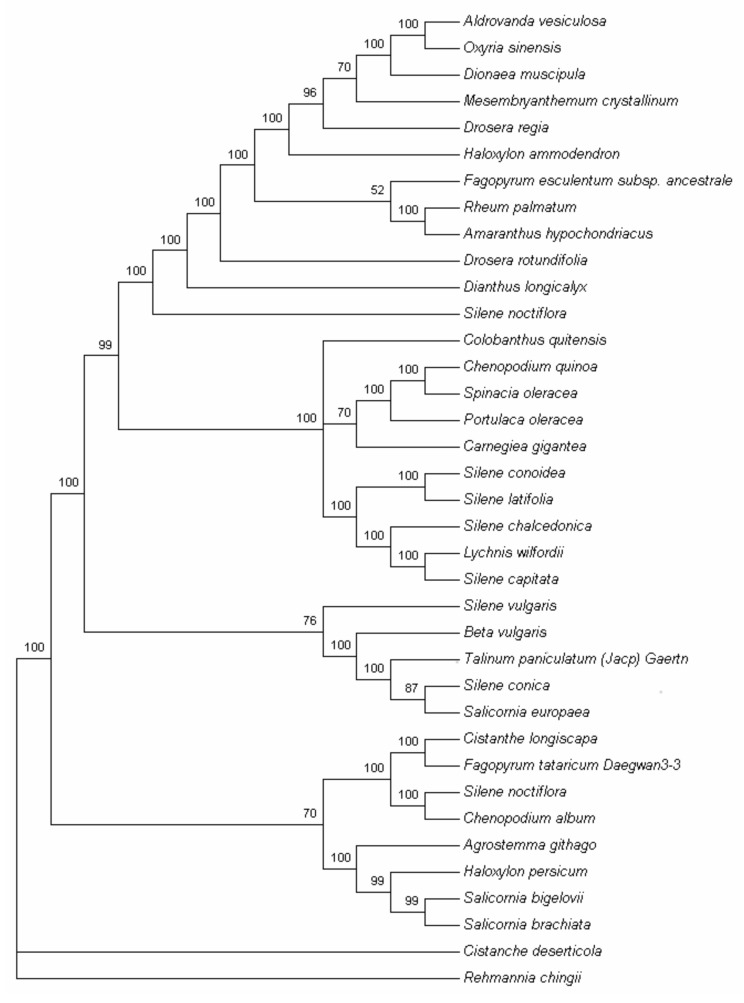
Phylogenetic tree of the 35 species in Caryophyllales using maximum parsimony (MP) and tree bisection-reconnection (TBR) analysis based on 48 protein-coding genes using a non-partitioning scheme. The phylogenetic tree was drawn using *Cistanche deserticola* and *Rehmannia chingiis* as outgroup.

The cp genome sequence is a useful resource for studying the taxonomy in the Angiosperm clade, and for analyzing evolutionary relationships within families. Here, to obtain a reasonable phylogenetic status of *T. paniculatum*, we performed multiple sequence alignments of cp genome protein coding genes. A total of 35 complete cp genomes of Caryophyllales and two outgroup species were subjected to phylogenetic analysis based on a 48-gene data matrix, which used the MP and ML methods. MP analysis resulted in a single tree with a length of 24,669, a consistency index (CI) of 0.6111, and a retention index (RI) of 0.7786 (Figure 8). Bootstrap analysis showed that 28 out of the 33 nodes had bootstrap values >95%.

As we all know, the specific relationships within the Poltulaca and Talinum remain obscure due to their complex past evolutionary histories. Recently, according to molecular and morphological evidence, the Anacampserotaceae, Basellaceae, Cactaceae, Didiereaceae, Portulacaceae, and Talinaceae were classified into four single independent families [17,18,19,20]. The Portulacaeae are now comprised only a single *Portulace* genus [21]. However, few reports have been published on the genetic diversity of chloroplast DNA from the Anacampserotaceae, Basellaceae, Cactaceae, Didiereaceae and Portulacaceae. From Figure 8, the cp genome protein coding genes of the phylogenetic trees show that *P. oleracea* and *T. paniculatum* do not belong to the same family, although the above data shows that there are many similarities between *P. oleracea* and *T. paniculatum*, which is consistent with the recent classification studies [20,45,46]. Hence, whole cp genome or the key segments of evolutionary variation should be used for phylogenetic study.

## 3. Discussion

We report a genome sequence of *T. paniculatum*, which provides an important resource for studying the evolution of the Caryophyllales and the molecular breeding of *T. paniculatum* with high pharmaceutical efficacy. Despite the fact that the chloroplast genomes of Angiosperms are well-conserved in the genomic structure in terms of gene order and number, length variations of the whole chloroplast genome sequences and LSC, SSC and IR regions, the IR expansion and contraction occur frequently. The results reported here are congruent with the recent studies which showed that the *trnH-GUG* gene was situated in the LSC region in some species of Caryophyllales, while the SSC/IRA border extends into the *ycf1* with subsequent formation of a *ycf1* pseudogene [47,48]. Boundary expansion and contraction between the single copy and IR boundary regions lead to sequence variation, which might be a base of plant lineages [49]. 

In this study, we analyzed codon usage frequency and RSCU in the *T. paniculatum*. As previously reported, leucine and isoleucine are the more commonly seen amino acids in the cp genomes of Angiosperms [33,50,51,52,53]. Likewise, like in earlier studies about repeats and SSRs, mononucleotide repeats are more abundant with A/T repeats, which is consistent with AT richness in Angiosperm chloroplast genomes [54,55,56]. Complement repeats were rarely found in *T. paniculatum* and *C. gigantea* [24]. Meanwhile, all of the SSRs in CDs locate in the LSC region [32]. These cp SSR markers could be a resource for molecular-marker-assisted selection breeding for *T. paniculatum* for production of high levels of biologically active compounds.

Here, we compared the *matK* and *rpl23* sequences of several species of Caryophyllales. The results revealed that the pseudogene *rpl23* of *T. paniculatum* had inserted AATT, which is different from other Caryophyllales. The gene of *matK* is often used to identity the relationship between species as a barcoding marker [57]. Most reports have revealed that the closer the relationship between species, the higher the sequence similarity of *matK*. *matK* represents one of the best choices for DNA barcoding, phylogenetic and evolutionary analysis [38,39,40]. However, though *T. paniculatum* has a high *matK* gene similarity to *P. oleracea*, they belong to different families, hence, multiple marks should be used for barcoding.

## 4. Conclusions 

In this study, the complete cp genome of *T. paniculatum* was reported and analyzed for the first time. *T. paniculatum* is one of the key traditional Chinese medicines used against cancer, diabetes, hepatic disorders, leishmaniasis and reproductive disorders and is also an edible vegetable. Comparing the cp genomes of *T. paniculatum* with other Caryophyllale species, the cp genome of *T. paniculatum* is the largest IRs, but has the smallest *rps19* and *ycf1* length in the IR border, most likely due to low occurrence of contraction by the fragment deletions in the intergenic regions. The pseudogene of *rpl23* was inserted by AATT, and *trnK-UUU* and *rpl16* have larger introns than other Caryophyllale species. The *matK* genes show rich divergence. All these results provide good opportunities for future barcoding molecular marker development. Our phylogenetic analysis showed that *T. paniculatum* and *P. oleracea* don’t belong to the same family. This information will be useful for the phylogenetic study of *T. paniculatum*, and might also contribute to the genetics and breeding of *T. paniculatum*.

## 5. Materials and Methods

### 5.1. DNA Sequencing and Genome Assembly

Total DNA of *T. paniculatum* was obtained from approximately 100 g of fresh leaves using the CTAB method [58]. Quality of the DNA was evaluated by measuring A_260_ using a Nanodrop2000 spectrometer (Thermo Fisher Scientific, Waltham, MA, USA). Then, the DNA was sheared to fragments of 300~500 bp. Paired-end libraries were prepared with the TruSeq^TM^ DNA sample Prep Kit and the TruSeq PE Cluster Kit. The genome was then sequenced using the HiSeq4000 platform (Illumina Inc., San Diego, CA, USA). The assembly of the cp genome of *T. paniculatum* was first carried out through the error correction and production of initial contigs using the GS FLX De Novo Assembler Software (Newbler V2.6). PCR amplification and Sanger sequencing were performed to verify the four junction regions between the IRs and the LSC/SSC. The final cp genome of *T. paniculatum* was submitted to GenBank with the accession number MG710385. 

### 5.2. Gene Annotation and Codon Usage Analysis

The cp genome was annotated by manual corrections using BLAST and DOGMA [59]. The tRNAscan-SE [60] was used to identify the tRNA genes. OGDRAW [61] was used to draw the circular genome map. MEGA5 were used for revealing the characteristics of the variations in synonymous codon usage [62]. The relative synonymous codon usage values (RSCU), codon usage and GC content were also determined by MEGA5. 

### 5.3. Repeat Structure and Single Sequence Repeats (SSRs) Analysis 

Analysis of tandem repeats with more than 30 bp and a minimum of 90% sequence (forward, palindromic, reverse and complement) and single sequence repeats (SSRs) was identified by REPuter [63] and MISA respectively, with the same parameters as described in Ni et al. [43].

### 5.4. Comparative Genome Analysis of the T. paniculatum with Eight cp Genomes of Caryophyllales 

Comparison of the overall cp genome of *T. paniculatum* with eight cp genomes of Caryophyllales were performed by mVISTA [64,65], using the annotation of *T. paniculatum* as a reference.

### 5.5. Phylogenetic Analysis

A total of 37 complete cp genome sequences were downloaded from the NCBI Organelle Genome Resources database (http://www.ncbi.nlm.nih.gov/genomes/). For the phylogenetic analysis, a set of 48 protein-coding genes that were common in the 37 analyzed genomes, was used. Maximum parsimony (MP) analysis was performed with PAUP*4.0b10 [66], using a heuristic search combined with the random addition of 1000 replicates and tree bisection-reconnection (TBR) branch swapping, in the Multrees option. Bootstrap analysis was also performed with 1000 replicates and TBR branch swapping. *Rehmannia chingii* and *Lindenbergia philippensis* were set as outgroups.

## Figures and Tables

**Table 1:** Chloroplast genome composition of *Talinum paniculatum* (*Jacp*) Gaertn.

Region	Size (bp)	T(U) (%)	C (%)	A (%)	G (%)	Genes	Protein-Coding Genes	tRNA Genes	rRNA Genes
LSC	86,898	33.2	17.8	32.2	16.8	83	56	22	0
SSC	18,529	34.6	15.9	34.9	14.6	12	12	1	0
IRA	25,751	29.0	22.3	28.2	20.4	19	7	7	4
IRB	25,751	28.2	20.4	29.0	22.3	19	8	7	4
Total	156,929	31.8	19.0	30.7	18.5	128	83 (4)	37 (7)	8 (4)
CDS	78,438	31.5	17.6	30.8	20.1				
1st position	26,146	23.7	18.8	30.7	26.7				
2nd position	26,146	32.7	20.2	29.4	17.8				
3rd position	26,146	38.1	13.7	32.4	15.7				

CDS: protein-coding regions. The numbers in brackets represent the number of repeated genes.

**Table 2:** Genes of the *Talinum paniculatum* (*Jacp*) Gaertn.

Group of Genes	Name of Gene	No.
Photosytem I	*psaA*, *psaB*, *psaC*, *psaI*, *psaJ*	5
Photosytem II	*psbA*, *psbB*, *psbC*, *psbD*, *psbE*, *psbF*, *psbH*, *psbI*, *psbJ*, *psbK*, *psbL*, *psbM*, *psbN*, *psbT*, *psbZ*	15
Cytochrome b/f complex	*petA*, *petB*, *petD*, *petG*, *petL*, *petN*	6
ATP system	*atpA*, *atpB*, *atpF*, *atpF*, *atpH*, *atpI*	6
NADH dehydrogenase	*ndhA*, *ndhB* *, *ndhC*, *ndhD*, *ndhE*, *ndhF*, *ndhG*, *ndhH*, *ndhI*, *ndhJ*, *ndhK*	12
RuBisCO large subnit	*rbcL*	1
RNA polymerase	*rpoA*, *rpoB*, *rpoC1*, *rpoC2*	4
Ribosomal proteins (SSU)	*rps2*, *rps3*, *rps4*, *rps7* *, *rps8*, *rps11*, *rps12* *, *rps14*, *rps15*, *rps16*, *rps18*, *rps19*	14
Ribosomal proteins (LSU)	*rpl2*, *rpl14*, *rpl16*, *rpl19*, *rpl20*, *rpl22*, *rpl32*, *rpl33*, *rpl36*	9
Miscellaneous proteins	*accD*, *cemA*, *ccsA*, *clpP*, *infA*, *matK*	6
Hypothetical chloroplast reading frames (ycf)	*ycf1*, *ycf2* *, *ycf3*, *ycf4*	5
Transfer RNAs	*trnA-UGC* *, *trnC-GCA*, *trnD-GUC*, *trnE-UUC*, *trnF-GAA*, *trnG-UCC*, *trnG-GCC*, *trnH-GUG*, *trnI-CAU* *, *trnIGAU* *, *trnK-UUU*, *trnL-UAA*, *trnL-CAA* *, *trnL-UAG*, *trnM-CAU*, *trnN-GUU* *, *trnP-UGG*, *trnQ-UUG*, *trnR-UCU*, *trnRACG* *, *trnS-GCU*, *trnS-UGA*, *trnS-GGA*, *trnT-GGU*, *trnT-UGU*, *trnV-UAC*, *trnV-GAC* *, *trnW-CCA*, *trnY-GUA*, *trnfM-CAU*	37
Ribosomal RNAs	*rrn4.5* *, *rrn5* *, *rrn16* *, *rrn23* *	8
Pseudogene	*rpl23* *, *rps19*, *ycf1*	4
Total		132

* indicates a duplicated gene.

**Table 3:** The intron-containing genes in the *T. paniculatum* cp genome and the lengths of the exons and introns.

No.	Gene	Location	ExonI (bp)	IntronI (bp)	ExonII (bp)	IntronII (bp)	ExonIII (bp)
1	*trnK-UUU*	LSC	35	2502	37		
2	*rps16*	LSC	202	867	41		
3	*trnG-UCC*	LSC	23	707	48		
4	*atpF*	LSC	410	745	145		
5	*rpoC1*	LSC	1611	794	432		
6	*ycf3*	LSC	153	773	229	769	125
7	*trnL-UAA*	LSC	37	599	50		
8	*trnV-UAC*	LSC	35	586	38		
9	*clpP*	LSC	229	590	291	894	71
10	*petB*	LSC	6	768	642		
11	*petD*	LSC	8	792	475		
12	*rpl16*	LSC	399	1102	9		
13	*ndhB* *	IR	756	668	777		
14	*rps12* *	IR	114	-	232	533	26
15	*trnI-GAU* *	IR	37	947	35		
16	*trnA-UGC* *	IR	38	818	35		
17	*ndhA*	SSC	539	1087	553		

* indicates duplicated gene.

**Table 4:** Codon usage in the *T. paniculatum* cp genome.

Amino Acid	Codon	Count	RSCU	Amino Acid	Codon	Count	RSCU	Amino Acid	Codon	Count	RSCU	Amino Acid	Codon	Count	RSCU
Phe	UUU(F)	975	1.3	Ser	UCU(S)	556	1.67	Tyr	UAU(Y)	779	1.61	Stop	UGA(*)	20	0.72
Phe	UUC(F)	527	0.7	Ser	UCC(S)	331	0.99	Tyr	UAC(Y)	190	0.39	Trp	UGG(W)	460	1
Leu	UUA(L)	854	1.84	Ser	UCA(S)	408	1.22	Stop	UAA(*)	46	1.66	Ala	GCU(A)	615	1.75
Leu	UUG(L)	554	1.2	Ser	UCG(S)	187	0.56	Stop	UAG(*)	17	0.61	Ala	GCC(A)	234	0.66
Leu	CUU(L)	601	1.3	Ser	AGU(S)	393	1.18	His	CAU(H)	448	1.49	Ala	GCA(A)	416	1.18
Leu	CUC(L)	179	0.39	Ser	AGC(S)	127	0.38	His	CAC(H)	155	0.51	Ala	GCG(A)	144	0.41
Leu	CUA(L)	408	0.88	Pro	CCU(P)	426	1.6	Gln	CAA(Q)	729	1.54	Arg	CGU(R)	360	1.36
Leu	CUG(L)	184	0.4	Pro	CCC(P)	200	0.75	Gln	CAG(Q)	216	0.46	Arg	CGC(R)	92	0.35
Ile	AUU(I)	1130	1.5	Pro	CCA(P)	299	1.12	Asn	AAU(N)	960	1.53	Arg	CGA(R)	366	1.38
Ile	AUC(I)	410	0.54	Pro	CCG(P)	143	0.54	Asn	AAC(N)	297	0.47	Arg	CGG(R)	120	0.45
Ile	AUA(I)	718	0.95	Thr	ACU(T)	531	1.61	Lys	AAA(K)	1061	1.5	Arg	AGA(R)	475	1.79
Met	AUG(M)	607	1	Thr	ACC(T)	250	0.76	Lys	AAG(K)	357	0.5	Arg	AGG(R)	175	0.66
Val	GUU(V)	514	1.48	Thr	ACA(T)	402	1.22	Asp	GAU(D)	883	1.66	Gly	GGU(G)	552	1.26
Val	GUC(V)	160	0.46	Thr	ACG(T)	134	0.41	Asp	GAC(D)	184	0.34	Gly	GGC(G)	190	0.43
Val	GUA(V)	522	1.5	Cys	UGU(C)	235	1.55	Glu	GAA(E)	1041	1.53	Gly	GGA(G)	712	1.62
Val	GUG(V)	194	0.56	Cys	UGC(C)	68	0.45	Glu	GAG(E)	323	0.47	Gly	GGG(G)	302	0.69

RSCU: Relative synonymous codon usage. RSCU > 1 are highlighted in bold. * indicates stop codon.

**Table 5:** Repeat sequences distribution in the *T. paniculatum* (Jacp) Gaertn chloroplast genome.

No.	Size (bp)	Type	Repeat 1 Start	Repeat 1 Location	Repeat 2 Start	Repeat 2 Location	Location
1	35	F	3144	matK	6441	IGS (*rps16*, *trnQ-UUG*)	LSC
2	30	P	4153	IGS (*trnK-UUU* (exon), *rps16*)	4211	IGS (*trnK-UUU* (exon), *rps16*)	LSC
3	30	R	4578	IGS (*trnK-UUU* (exon), *rps16*)	4581	IGS (*trnK-UUU* (exon), *rps16*)	LSC
4	30	C	4581	IGS (*trnK-UUU* (exon), *rps16*)	4582	IGS (*trnK-UUU* (exon), *rps16*)	LSC
5	30	R	6862	IGS (*trnQ-UUG*, *psbK*)	8268	IGS (*trnS-GCU*, *trnG-UCC*)	LSC
6	30	F	7743	IGS (*psbI*, *trnS-GCU*)	7770	IGS (*psbI*, *trnS-GCU*)	LSC
7	32	F	7896	IGS (*psbI*, *trnS-GCU*)	36,017	IGS (*psbC*, *trnS-UGA*)	LSC
8	30	P	7898	IGS (*psbI*, *trnS-GCU*)	46,268	*trnS-GGA*	LSC
9	37	R	8258	IGS (*trnS-GCU*, *trnG-UCC*)	8261	IGS (*trnS-GCU*, *trnG-UCC*)	LSC
10	37	R	8258	IGS (*trnS-GCU*, *trnG-UCC*)	8264	IGS (*trnS-GCU*, *trnG-UCC*)	LSC
11	35	F	8258	IGS (*trnS-GCU*, *trnG-UCC*)	8277	IGS (*trnS-GCU*, *trnG-UCC*)	LSC
12	35	R	8266	IGS (*trnS-GCU*, *trnG-UCC*)	8277	IGS (trnS-GCU, *trnG-UCC*)	LSC
13	34	R	8258	IGS (*trnS-GCU*, *trnG-UCC*)	8261	IGS (trnS-GCU, *trnG-UCC*)	LSC
14	33	F	8261	IGS (*trnS-GCU*, *trnG-UCC*)	8283	IGS (*trnS-GCU*, *trnG-UCC*)	LSC
15	32	R	8263	IGS (*trnS-GCU*, *trnG-UCC*)	8280	IGS (*trnS-GCU*, *trnG-UCC*)	LSC
16	31	F	8261	IGS (*trnS-GCU*, *trnG-UCC*)	8264	IGS (*trnS-GCU*, *trnG-UCC*)	LSC
17	31	R	8261	IGS (*trnS-GCU*, *trnG-UCC*)	8280	IGS (*trnS-GCU*, *trnG-UCC*)	LSC
18	31	R	8267	IGS (*trnS-GCU*, *trnG-UCC*)	29,873	IGS (*psbM*, *trnD-GUC*)	LSC
19	30	P	8267	IGS (*trnS-GCU*, *trnG-UCC*)	62,668	IGS (*ycf4*, *cemA*)	LSC
20	30	P	8280	IGS (*trnS-GCU*, *trnG-UCC*)	31,428	IGS (*trnE-UUC*, *trnT-GGU*)	LSC
21	31	F	9566	*trnG-UCC*	37,057	*trnG-GCC*	LSC
22	30	P	36,019	IGS (*psbC*, *trnS-UGA*)	46,268	*trnS-GGA*	LSC
23	30	F	39,314	*psaB*	41,538	*psaA*	LSC
24	42	F	44,540	*ycf3* (intronII)	123,558	*ndhA* (intron)	LSC, SSC
25	39	F	44,543	*ycf3* (intronII)	100,738	IGS (*rps12*, *trnV-GAC*)	LSC, IRb
26	39	P	44,543	*ycf3* (intronII)	143,050	IGS (*trnV-GAC*, rps12)	LSC, IRa
27	30	F	44,555	*ycf3* (intronII)	100,750	IGS (*rps12*, *trnV-GAC*)	LSC, IRb
28	30	P	44,555	*ycf3* (intronII)	143,047	IGS (*trnV-GAC*, *rps12*)	LSC, IRa
29	40	P	76,849	IGS (*psbT*, *psbN*)	76,849	IGS (*psbT*, *psbN*)	LSC
30	30	P	84,344	IGS (*trnS-GCU*, *trnG-UCC*)	84,346	IGS (*trnS-GCU*, *trnG-UCC*)	LSC
31	61	F	93,517	*ycf2*	93,535	*ycf2*	IRb
32	61	P	93,517	*ycf2*	150,231	*ycf2*	IRb, IRa
33	61	P	93,535	*ycf2*	150,249	*ycf2*	IRb, IRa
34	61	F	150,231	*ycf2*	150,249	*ycf2*	IRa
35	52	F	93,526	*ycf2*	93,544	*ycf2*	IRb
36	52	P	93,526	*ycf2*	150,231	*ycf2*	IRb, IRa
37	52	P	93,544	*ycf2*	150,249	*ycf2*	IRb, IRa
38	34	F	93,526	*ycf2*	93,562	*ycf2*	IRb
39	34	P	93,526	*ycf2*	150,231	*ycf2*	IRb, IRa
40	34	P	93,562	*ycf2*	150,267	*ycf2*	IRb, IRa
41	43	F	93,517	*ycf2*	93,533	*ycf2*	IRb
42	43	P	93,517	*ycf2*	150,231	*ycf2*	IRb, IRa
43	43	P	93,553	*ycf2*	150,267	*ycf2*	IRb, IRa
44	43	F	150,231	*ycf2*	150,267	*ycf2*	IRa
45	40	F	100,738	IGS (*rps12*, *trnV-GAC*)	123,561	*ndhA* (intron)	IRb, SSC
46	34	F	109,506	IGS (*rrn4.5*, *rrn5*)	109,538	IGS (*rrn4.5*, *rrn5*)	IRb
47	34	F	109,506	IGS (*rrn4.5*, *rrn5*)	134,255	IGS (*rrn5*, *rrn4.5*)	IRb, IRa
48	34	P	109,538	IGS (*rrn4.5*, *rrn5*)	134,287	IGS (*rrn5*, *rrn4.5*)	IRb, IRa
49	34	P	134,255	IGS (*rrn5*, *rrn4.5*)	134,287	IGS (*rrn5*, *rrn4.5*)	IRa
50	38	P	118,646	IGS (*ccsA*, *ndhD*)	118,646	IGS (*ccsA*, *ndhD*)	SSC
51	40	P	123,561	*ndhA* (intron)	143,049	IGS(*trnV-GAC*, *rps12*)	SSC, IRa

F: forward repeat; P: palindrome (inverted) repeat; R: reverse repeat; C: complement repeat. IGS: intergenic spacer.

**Table 6:** Frequency of simple sequence repeats in the *T. paniculatum* chloroplast genome.

Length Unit	10	10	11	12	13	14	15	16	17	18	19	20	Total
A	15	15	3	8	1	1	3			1	1		33
T	14	14	4	7	3	2	1		1		1		33
C	1	1											1
G	1	1											1
AG	1	1											1
AT	4	4		3									7
TA	2	2		1				1					4
AAT	1	1											1
ATA				1									1
TTA				2									2
TAT				1									1
AGGT				1									1
ATGG				1									1
AATT				1									1
CTAC				1									1
TTTC				1									1
TAAT				1									1
GGAA				1									1

**Table 7:** Simple sequence repeats in the CDSs of the *T. paniculatum* chloroplast genome.

No.	Type	Motif	Size	Start	End	Location	Region
1	P1	(A)10	10	47	56	*trnH-UGG*	LSC
2	P1	(A)10	10	637	646	*psbA*	LSC
3	P1	(A)11	11	2104	2114	*matK*	LSC
4	P1	(A)12	12	3942	3953	*trnK-UUU* (intron)	LSC
5	P2	(AT)5	10	755	764	*psbA*	LSC
6	P4	(AATT)3	12	3974	3985	*trnK-UUU* (intron)	LSC
7	P4	(CCAT)3	12	54	65	*trnH-UGG*	LSC
